# Dental Teachings

**Published:** 1860-10

**Authors:** W. H. Atkinson


### Original Essays and Communications.

### DENTAL TEACHINGS.

BY W. H. ATKINSON.

Read before the American Dental Convention at Saratoga, August 9th, 1860.

ALL teaching must needs flow to the mind from a plane above it, or it is but a repetition of knowledge already attained, and if not forgotten, ready for *use*.

Had our teachings been *entire*, instead of horribly fragmentary, such aphorisms as the above would have been uncalled for.

*Feeding* seems to be the main business to which tooth doctors should direct their attention. Just how to define feeding is the great stumbling-block in the way of those who would help folks to a sound body.

Science being a circle, it matters but little at what point in that circle we make our start. Nevertheless, there are certain divisions in this circle, that it will facilitate progress to note, and begin at the line dividing them, and then travel either way to complete the curriculum.

And as "Dental Science," in the very nature of things, is but a small division of general science, we will do well to take heed that we keep within the legitimate limits of that department.

*Feeding* properly belongs to cell, organ and system; and that the organs and cells constituting our systems may be fed, pabulum must first be brought into the system, and *there prepared* for the purposes of nutrition, which always takes place in cells first, and these, by developing organs by aggregations of these cells, growth and nutrition are effected. So that feeding, in the strictest sense, belongs to that little understood part of our being, its basal and elemental existences. But in the sense of supply to the system, feeding takes place in mouths, in which TEETH are the principal means of division and comminution; then, that we may be *well* fed, it becomes necessary for *these* to be in sound and regular condition, to secure which is the object of all dental teachings.

Now, if the first proposition, aphorism or statement be true, we do not stand fully up (as individuals) to our aggregate advancement in every department of our knowledge, and hence some are on a plane above their fellows in some things, and they on a plane above us in others. So if our teachings and learnings have been fractional, we are, of course, not fit to lead in all departments.

To properly understand our relative positions, we must come in contact, compare notes; and this is best effected, not on a logical basis of argumentation, or long, prosy dissertations upon the details of our specialty of medical science, but by the free interchange of untrammeled questioning, in a word, practical and scientific conversations.

If any object and say, "no man *can* do as well at an off-hand, prompt answering of queries upon the moment," I have only to refer him to the explanatory note after the first aphorism in this paper, to show him where he stands—which convicts him of either never having known the answer, or else having been so little interested in it as to permit it to be forgotten, and really gives us the best measure of the man. For who among us, if a sufficient time for research be allowed him, is not capable of collecting all that has ever been written on any given question by the shrewdest minds and most laborious hands—and thus steal the real thunder of some one else, to flash forth its lightning, to illuminate our worthless heads with the halo of a temporary glory, and thus entrap us into the error of hoping to find a reservoir of all the knowledge treated of in the paper, in the head of the writer, only to be let down to zero when the proof of a few pointed questions are directly and well put!

It is, gentlemen, depend upon it, only the offspring of *untruth* and a desire to pass for more than we are worth, that first inaugurated this mode of doing up scientific discussions on this side of the great water. An ambition to lead has taken hold of the great majority of our teachers in the various Colleges and Academies of scientific lore. And from this starting point they have traveled a certain way and stuck their stakes, (boldly as ignorantly) asserting that all beyond was in the "unknowable" regions of dark nonentity. If you wish a certain proof of my assertion, please just retort back the same inquisition upon the "Leader," that he unfeelingly put upon his pupil, and his reply will clearly define where he is. If he patiently goes through a clear and satisfactorily elucidated explanation, he is a leader, and worthy so to rank. But if he throws himself back upon his dignity, and measuredly says, "My dear sir, I wish you to know *you* can not quiz *me*," then he is, beyond all question, *not* a legitimate leader or teacher.

Probably no one, during his whole career of instructing, has been so pure and truthful as not (at some period or incident of his experience) to see his own portrait in this odious picture. But to those who are willing and determined to advance to the point where they will be able to stand the *test*, I would say, you need not tarry in all the plain of sloth and error, but with the fleetness of the antelope flee to the pure elevations of the mountain fastnesses of science and truth, where the bare remembrance of former sins and trials shall only enhance your joy at being fully delivered from them, and being introduced to the blessed freedom that a knowledge of the truth alone can give.

Then let us not imprison ourselves in false dignity and puerile conceptions, while the whole field of pure science and research lies open before us, inviting us to the rich culminations of her varied branches, in a truly advanced and useful practice of a *living*, if not "well established" profession.

And for the encouragement of those members of our specialty who have thought themselves the very men with whom knowledge was doomed to die, I cheerfully reiterate, "Where *sin* abounded, *grace* did much *more* abound!" And "whosoever will, let him come and *partake*" of the light *beginning* to focalize in the congregating of the most earnest of our body, from time to time, to illuminate the whole by the aggregation of each one's little spark, until, by the laws of affinity and impartation, we shall have equipoised the entire of what the mass possesses so perfectly as to have banished the word "failed" from our vocabulary.

Let each one freely open his mouth, and not be frightened at his own noise, as Crusoe was, but persistently keep it opened till it is filled with something to SAY, and then truthfully and plainly project it, and I will warrant we will have a profitable time, especially if we follow the simple conversational plan. After each speaker has given vent to his pent up goods (that have laid musting on his mental shelves so long, that he forgot to eject them, if for no other purpose, to make place for new and more appropriate ones for the time and occasion), let any and all be free to propound questions to him touching the subject-matter of his speech.

In this way the ball will be at once easily set to rolling, and you who do not like to see it move, had better get out of the way; for these conditions fulfilled, such a rate of progress will have been secured as will be a caution to all who like the old ox-team style.

The advantages resulting from such a course are too patent and numerous to require specific statement. I therefore will only name a few.

I. Freedom from dignity and restraint.

II. Fraternity and unity of *feeling* (the highest of our faculties.) We ought to come together, not to manufacture, but exchange commodities, as at Irish and other fairs. And he who has come only to buy for the ready cash of attentive, intelligent listening, will probably go away quite as well satisfied as the cask just ready to burst for freedom, will after having ventilated itself to its perfect easing, only to be replenished with new material upon which to exert its refining power of fermentation, till the new contents shall have brought it to the declamatory state by full ripeness, and they in turn be freed by a like explosion.

With something like the foregoing, with the following for a chart or tressel-board upon which to draw out designs :

I. Be good and true.

II. So use thine own as not to infringe the right of thy fellow.

III. Do good and *no* harm all the days of thy life.

IV. When rebuked, count it a joy and evidence of preparation for the rebuker or rebuked to be advanced.

V. "Let brotherly love continue."

Now, after thus disturbing, I cheerfully and hopefully await the *moving* of the waters.

